# Brain Metastases in Soft Tissue Sarcomas: Case Report and Literature Review

**DOI:** 10.1080/13577140500190921

**Published:** 2005

**Authors:** Tejpal Gupta, Siddhartha Laskar, Sumeet Gujral, Mary Ann Muckaden

**Affiliations:** 1 Tata Memorial Centre Institute Tata Memorial Centre Mumbai India; 2 Department of Radiation Oncology Clinical Research Centre Advanced Centre for Treatment Research & Education in Cancer (ACTREC) Tata Memorial Centre, Institute Tata Memorial Centre, Kharghar, Navi Mumbai Mumbai 410208 India

## Abstract

*Background and purpose:* Brain metastasis is a relatively uncommon event in the natural history of soft tissue sarcomas. The increasing use of chemotherapy may have caused a reduction in local relapses as well as distant failures leading to an improvement in survival, thereby allowing metachronous seeding of the brain, a sanctuary site. The purpose of this report is to increase awareness amongst clinicians regarding such a possibility.

*Patients and methods: *A review of the departmental sarcoma database following the presentation of this index case in the clinic.

*Results and discussion:* An adolescent male who had previously been treated with surgery and radiotherapy for a spindle cell sarcoma of the left thigh developed a space-occupying lesion in the brain within 6 months of treatment of the primary tumor. He subsequently underwent resection of the presumed solitary brain metastasis followed by whole brain radiotherapy. On radiation he was detected to have pulmonary metastases too, for which he was offered palliative chemotherapy. The patient died of brain metastasis within 4 months. A review of the departmental sarcoma database, restricted to soft tissue sarcomas purely, maintained prospectively from 2000 till date, could not identify any other such case.

*Conclusion:* Brain metastases from soft tissue sarcomas are rare. Patients with neurological symptoms, however, should be appropriately investigated. Surgical resection of brain metastasis could be considered for solitary brain metastasis in non-eloquent areas. Palliative radiotherapy is appropriate for patients with multiple brain metastases or co-existing extra-cranial disease.


*Sarcoma*, September/December 2005; 9(3/4): 147–150

Taylor & Francis

Taylor & Francis Group

**CASE REPORT**

### Brain metastases in soft tissue sarcomas: Case report and literature review

TEJPAL GUPTA, SIDDHARTHA LASKAR, SUMEET GUJRAL, & MARY ANN MUCKADEN

*Tata Memorial Centre, Institute Tata Memorial Centre, Mumbai, India*

**Abstract**

*Background and purpose:* Brain metastasis is a relatively uncommon event in the natural history of soft tissue sarcomas. The increasing use of chemotherapy may have caused a reduction in local relapses as well as distant failures leading to an improvement in survival, thereby allowing metachronous seeding of the brain, a sanctuary site. The purpose of this report is to increase awareness amongst clinicians regarding such a possibility.

*Patients and methods:* A review of the departmental sarcoma database following the presentation of this index case in the clinic.

*Results and discussion:* An adolescent male who had previously been treated with surgery and radiotherapy for a spindle cell sarcoma of the left thigh developed a space-occupying lesion in the brain within 6 months of treatment of the primary tumor. He subsequently underwent resection of the presumed solitary brain metastasis followed by whole brain radiotherapy. On radiation he was detected to have pulmonary metastases too, for which he was offered palliative chemotherapy. The patient died of brain metastasis within 4 months. A review of the departmental sarcoma database, restricted to soft tissue sarcomas purely, maintained prospectively from 2000 till date, could not identify any other such case.

*Conclusion:* Brain metastases from soft tissue sarcomas are rare. Patients with neurological symptoms, however, should be appropriately investigated. Surgical resection of brain metastasis could be considered for solitary brain metastasis in non-eloquent areas. Palliative radiotherapy is appropriate for patients with multiple brain metastases or co-existing extra-cranial disease.

**Keywords:** *Brain metastases, pulmonary metastases, soft tissue sarcomas*

### Background and purpose

Brain metastasis is a relatively uncommon event in the natural history of soft tissue sarcomas (STS), more so in the absence of pulmonary metastases. In contrast, osteosarcomas, Ewing's sarcomas, primitive neuro-ectodermal tumors (PNETs), round cell sarcomas (rhabdomyosarcomas) and alveolar soft part sarcomas (ASPS) have a relatively increased propensity to metastasize to the brain. The increasing use of systemic chemotherapy in high-grade STS has improved outcome. It is associated with a significant reduction in local relapses as well as distant metastases. This may have led to a consequential improvement in disease-free survival and overall survival. This increase in survival could potentially provide an opportunity for metachronous seeding of the brain, a pharmacological sanctuary site [1]. The authors report on one such rare occurrence. The purpose of this report is to increase the awareness amongst clinicians regarding the possibility of such an occurrence and conduct a review of the sarcoma database maintained prospectively in the Department of Radiation Oncology since 2000.

### Patient and methods

Since the presentation of this index case, a review of the departmental sarcoma database was

Correspondence: Dr Tejpal Gupta, MD, DNB, Department of Radiation Oncology, Clinical Research Centre, Advanced Centre for Treatment Research & Education in Cancer (ACTREC), Tata Memorial Centre, Kharghar, Navi Mumbai, 410208, India. Tel: 91 22 27405057. Fax: 91 22 27412894. E-mail: tejpalgupta@rediffmail.com

ISSN 1357–714X print/ISSN 1369–1643 online/05/(03-04)0147-4 © 2005 Taylor & Francis
DOI: 10.1080/13577140500190921

carried out. Since 2000 to date, patients with non-rhabdomyosarcoma STS are being registered prospectively in a database maintained in the Department of Radiation Oncology. The medical records of all such registered patients were searched electronically. Patients with soft tissue sarcomas alone (excluding extra-skeletal Ewing's family of tumors, and sarcomas of uncertain histogenesis such as ASPS) were selected from the database and their case files retrieved to document the occurrence of brain metastases.

### Results

### *Case report*

An 18-year-old boy presented with complaints of recent onset headache and vomiting. These central nervous system (CNS) symptoms prompted a contrast-enhanced computed tomography (CECT) of the brain, which revealed a heterogenously enhancing mixed density space-occupying lesion in the right temporo-parietal region with moderate perifocal edema and mass effect with midline shift (Figure 1). Six months prior the patient had undergone wide excision of a soft tissue tumor of the left thigh that was reported as a pleomorphic spindle cell sarcoma of low to intermediate grade. He had also received postoperative adjuvant radiation to the tumor bed to a dose of 60 Gy/30 fractions/6 weeks and was locally controlled. In view of the antecedent history, the diagnosis of a solitary brain metastasis was favored. Until that time there was no evidence of pulmonary metastases on a chest radiograph (CXR). The patient underwent right temporal craniotomy with gross total resection of the lesion. Histopathology revealed it to be a metastatic high-grade spindle cell sarcoma consistent with a known primary STS. Slide review of the previous surgery (wide excision of the left thigh swelling) established that the lesion in the brain was indeed metastatic from that primary. Subsequently the patient was treated with palliative whole brain radiotherapy (WBRT) to a dose of 30 Gy/10 fractions/2 weeks with parallel opposed portals on a telecobalt unit. While on radiation he developed a cough for which he was adequately investigated. Although the CXR was still negative, a CECT of the thorax showed multiple lung nodules suggestive of bilateral pulmonary metastases. He was offered palliative chemotherapy that he declined. Subsequently the patient died of disseminated disease within 4 months of the diagnosis of brain metastases.

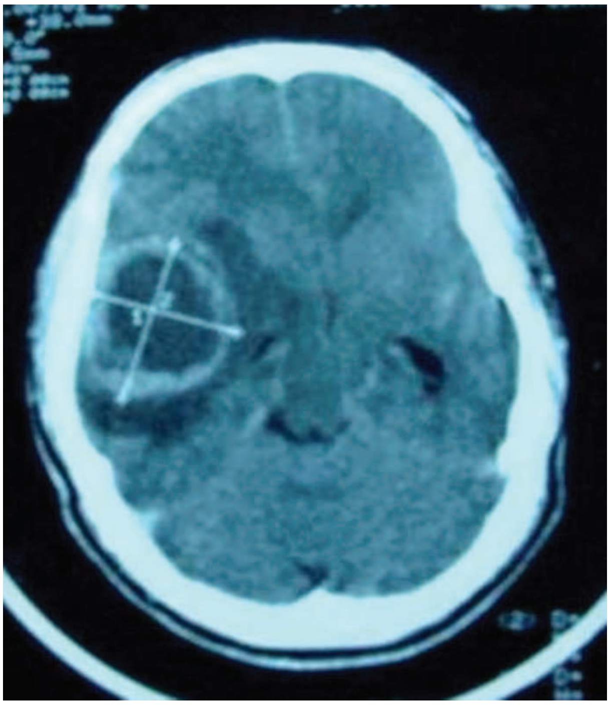

Figure 1. CT scan of the brain showing a heterogenously enhancing mixed density SOL in right temporo-parietal region with moderate perilesional edema and mass effect.

### *Database review*

The departmental sarcoma database had prospectively registered 368 patients with non-rhabdomyosarcoma STS from 2000 to date. After excluding extra-skeletal Ewing's family of tumors and ASPS (higher propensity to involve the brain), 307 patients with pure soft tissue sarcomas were identified. None of these 307 patients with pure STS, excepting the index patient described in this report had a documented brain metastasis on retrospective case file review.

### Discussion

The prognosis of patients with brain metastases is generally poor, the median survival of untreated patients being 1–2 months. The overall median survival increases to 4–6 months with therapy. Neurosurgical resection of a solitary brain metastasis is a good palliative procedure with low morbidity in experienced hands. It is also associated with better intra-cranial control and potentially improved survival. However, its role in multiple brain metastases or in patients with co-existing extra-cranial disease remains largely speculative. Given the resource implications and the questionable efficacy, open craniotomy could possibly be avoided in such setting and palliative radiotherapy offered to these patients with limited life expectancy.

Brain metastasis is thought to be a relatively uncommon event in the natural history of STS, particularly in the absence of pulmonary metastases. It is highly likely that this patient had asymptomatic pulmonary metastases prior to the

Table I. Selected series of brain metastases in sarcomas/pediatric solid tumors.

| Author [Ref.] | Total no. of patients (*N*) | Patients detected with brain metastases | Common histologies | Extracranial metastases | MS (months) |
|---|---|---|---|---|---|
| Postovsky et al. [4] | 411 | 14/236 (bone sarcomas) 4/175 (STS) | Ewings sarcoma, Osteosarcoma | 8/18 | 5 |
| Paulino et al. [5] | 611 | 30 | Neuroblastoma, RMS, Ewing's | 29/30 | 4 |
| Ogose et al. [6] | 480 | 15/268 (STS) 5/212 (bone sarcomas) | ASPS, Ewing's, RMS | 16/30 | 5.1 |
| Bindal et al. [7] | – | 21 | Osteosarcoma, Leiomyosarcoma | Not known | 11.8 |
| Wronski et al. [8] | 670 | 25 | Osteosarcoma, Embryonal RMS | 19/25 | 7 |
| Salvati et al. [9] | – | 15 | Osteosarcoma, Leiomyosarcoma | 9/15 | 9.3 |
| Espat et al. [10] | 3829 | 40 | Leiomyosarcoma, Liposarcoma, RMS | 18/19 | 7 |

MS, median survival; STS, soft tissue sarcomas; ASPS, alveolar soft part sarcoma; RMS, rhabdomyosarcoma.

development of brain metastasis, which was missed on a CXR. Had a CECT thorax been done instead of CXR, one would have been more circumspect in going ahead with a major neurosurgical procedure in the setting of disseminated lung metastases. The true incidence of symptomatic brain metastases in STS is not clearly known. It occurs in a small minority with a reported prevalence of 1–6%. The available literature is limited to autopsy series [2,3], few retrospective studies [4–6], small surgical series [7–9] and one recently published prospective study [10] (Table I). The issue is confounded by the fact that most of published series combine skeletal with extra-skeletal sarcomas, and round cell sarcomas (e.g., rhabdomyosarcomas or Ewing's sarcomas) with non-round cell sarcomas. The surgical case series suffer from an inherent selection bias.

In a retrospective review of 411 patients with various types of sarcomas, Postovsky et al. [4] identified 18 patients with brain metastases, an overall incidence of 4.3%. Soft tissue tumors had lesser (2.3%) CNS involvement as compared to bone sarcomas (5.9%), with Ewing's sarcoma being the most common. All these 18 patients with brain metastases had some neurological symptoms for which they were investigated appropriately. The management of brain metastatic disease was individualized and consisted of surgery, radiotherapy, chemotherapy, best supportive care or a combination of the above depending upon the general condition of the patient. The outcome of these patients was particularly bad with a median survival of 5 months from the diagnosis of brain metastases, despite aggressive therapy. Paulino and colleagues [5] reported a 4.9% incidence of brain metastases (30 patients) in pediatric solid tumors from the medical records of 611 children treated at University of Iowa Hospital over a 35-year period. Neuroblastoma (8%), rhabdomyosarcoma (6.7%), Ewing's sarcoma (5.7%) and osteosarcoma (4.7%) had a higher incidence of CNS metastases as compared to non-round cell soft tissue sarcoma (2.4%) and Wilm's tumor (1%). Of these 30 children, four were diagnosed to have brain metastases only at autopsy, whereas the remaining 26 non-autopsy patients had CNS symptoms which prompted neuro-imaging for radiological confirmation. Majority of the patients had concurrent distant disease either in the form of lung or bone metastases. The treatment strategy was very varied for these patients and the median survival after diagnosis of brain metastases was only 4 months with a 6-month and 1-year survival of 27 and 11.5%, respectively. On multivariate analysis, use of radiotherapy was the only factor that was associated with an improved freedom from neurological progression ($P = 0.005$) and a trend towards improved overall survival ($P = 0.07$). Ogose et al. [6], in a retrospective review of their institutional sarcoma database, identified 20 cases of symptomatic brain metastases from 480 patients treated and followed-up for at least a year (4.2%). Of these, 15 cases of brain metastases were recorded from 268 soft tissue tumors and five cases from 212 bone sarcomas. ASPS (3/4), extra-skeletal Ewing's (2/8), and rhabdomyosarcoma (2/13) tended to show a relatively high incidence of CNS involvement. The majority (16/20) of these patients had pulmonary metastases too. These patients were variably treated with surgical resection, WBRT, palliative chemotherapy or best supportive care with the mean survival after the diagnosis of brain metastases being 5.1 months.

Bindal et al. [7] reported on a series of 21 patients treated with surgical resection of intraparenchymal brain metastases from osteosarcoma (seven patients), leiomyosarcoma (four patients), malignant fibrous histiocytoma (three patients), alveolar soft part sarcoma (two patients), Ewing's sarcoma (three patients), and unclassified sarcomas (two patients). The median survival of the entire group after craniotomy was 11.8 months with pre-operative performance status and completeness of resection being the only factors associated with outcome. The presence of lung metastases did not impact upon survival and the authors concluded that it should not be regarded as a contra-indication to brain metastasectomy. In another surgical series, Wronski et al. [8] reported a 4% prevalence of sarcoma brain metastases (25 patients) in a group of 670 patients undergoing brain metastasectomy. Osteosarcomas and embryonal rhabdomyosarcomas were the commonest tumors of skeletal and non-skeletal origin, respectively, associated with brain metastases. Pulmonary metastases were present in the majority (19/25) of these patients, 12 of whom underwent pulmonary metastasectomy also in addition to the resection of brain metastases. The median survival post-craniotomy was 7 months with a 2-year estimated overall survival of 16%. The authors were unable to identify an association between improved survival for patients undergoing surgical resection of brain metastases and previous pulmonary metastasectomy. Salvati et al. [9] reported on 15 patients surgically treated for brain metastases from sarcoma, including six osteosarcomas, five leiomyosarcomas, two malignant fibrous histiocytomas, and two alveolar soft part sarcomas. The median survival of the entire group was 9.3 months following craniotomy. Performance status was the only factor significantly impacting upon outcome (median survival of 12.8 months in good performance status patients versus 5.3 months in poor performance status).

In the only prospective study cohort, Espat et al. [10] could identify 40 patients with brain metastases from a total of 3829 STS patients (1%) registered in a prospective database over a 20-year period. The most frequent subtype of STS metastasizing to the brain was leiomyosarcoma (eight patients), liposarcoma (five patients), rhabdomyosarcomas (four patients) and malignant fibrous histiocytomas (four patients). Of the 19 patients who developed brain metastases metachronously, 18 had lung metastases as the immediate prior site of disease. Such high frequency of pulmonary metastases is consistent with the preponderance of truncal or extremity sarcomas in this cohort. The first site of distant metastases for truncal or extremity STS is mostly the lung. In contrast patients with retroperitoneal or visceral STS die of local failure rather than surviving to develop distant metastases. Brain metastasectomy was done in 27 of the 40 patients and was significantly associated with the initial site of disease; 20 of the 27 patients who underwent resection versus two of the 13 patients not undergoing resection had extremity or truncal STS ($P < 0.001$). There was no association between parenchymal versus leptomeningeal site of metastases and any outcome measure. The 1- and 2-year disease-specific survival for the entire cohort of 40 patients was 55 and 25%, respectively, with a median survival of 15 months. The 1- and 2-year post-metastases survival rates were 34 and 20%, respectively, with a median survival of 7 months. Surgical resection of metastasis was associated with an improvement in survival (9.6 vs. 2.7 months for unresected patients, $p < 0.01$) and the authors concluded that metastasectomy may be appropriate treatment for selected patients with oligo-metastases.

### Conclusion

Although brain metastases from STS are rare, symptomatic patients should be appropriately investigated. Neurosurgical resection may be considered for solitary brain metastasis confined to non-eloquent cortex associated with significant mass effect. WBRT should be appropriate palliative treatment for patients with multiple brain metastases or co-existing extra-cranial disease. CECT thorax is needed to rule out pulmonary metastases definitely. In case of synchronous lung metastases, palliative chemotherapy may be offered. The outlook of these patients continues to remain grim despite aggressive therapy.

### Conflict of interest

None declared

### References

1. Espana P, Chang P, Wiernik PH. Increased incidence of brain metastases in sarcoma patients. Cancer 1980; 45(2):377–380.
2. Aronson SM, Garcia JH, Aronson BE. Metastatic neoplasms of the brain: Their frequency in relation to age. Cancer 1964;17:558–563.
3. Posner JB, Chernik NL. Intracranial metastases from systemic cancer. Adv Neurol 1978;19:579–592.
4. Postovsky S, Ash S, Ramu IN, Yaniv Y, Zaizov R, Futerman B, Elhasid R, Ben Barak A, Halil A, Ben Arush AW. Central nervous system involvement in children with sarcoma. Oncology 2003;65:118–124.
5. Paulino AC, Nguyen T, Barker JL Jr. Brain metastases in children with sarcoma, neuroblastoma and Wilm's tumor. Int J Radiat Oncol Biol Phys 2003;57(1):177–183.
6. Ogose A, Morita T, Hotta T, Kobayashi H, Otsuka H, Hirata Y, Yoshida S. Brain metastases in musculoskeletal sarcomas. Jpn J Clin Oncol 1999;29(5):245–247.
7. Bindal RK, Sawaya RE, Leavens ME, Taylor SH, Guinee VF. Sarcoma metastatic to the brain: Results of surgical treatment. Neurosurgery 1994;35:185–190.
8. Wronski M, Arbit E, Burt M, Perino G, Galicich JH, Brennan MF. Resection of brain metastases from sarcoma. Ann Surg Oncol 1995;2:392–395.
9. Salvati M, Cervoni L, Caruso R, Gagliardi FM, Delfini R. Sarcoma metastatic to the brain: A series of 15 cases. Surg Neurol 1998;49:441–444.
10. Espat JN, Bilsky M, Lewis JJ, Leung D, Brennan MF. Soft tissue sarcoma brain metastases: Prevalence in a cohort of 3829 patients. Cancer 2002;94:2706–2711.

